# The effect of Cissus quadrangularis (CQR-300) and a Cissus formulation (CORE) on obesity and obesity-induced oxidative stress

**DOI:** 10.1186/1476-511X-6-4

**Published:** 2007-02-04

**Authors:** Julius E Oben, Damaris Mandob Enyegue, Gilles I Fomekong, Yves B Soukontoua, Gabriel A Agbor

**Affiliations:** 1 Laboratory of Nutrition and Nutritional Biochemistry, Department of Biochemistry, B.P. 812, University of Yaoundé I, Yaoundé, Cameroon; 2 CRPMT, Institute of Medical Research and Medicinal Plants Studies, Yaoundé, Cameroon

## Abstract

**Aim:**

Obesity is generally linked to complications in lipid metabolism and oxidative stress. The aim of this study was to compare the effect of a proprietary extract of *Cissus quadrangularis *(CQR-300) to that of a proprietary formulation containing CQR-300 (CORE) on weight, blood lipids, and oxidative stress in overweight and obese people.

**Methods:**

The first part of the study investigated the *in vitro *antioxidant properties of CQR-300 and CORE using 3 different methods, while the second part of the study was a double-blind placebo controlled design, involving initially 168 overweight and obese persons (38.7% males; 61.3% females; ages 19–54), of whom 153 completed the study. All participants received two daily doses of CQR-300, CORE, or placebo and were encouraged to maintain their normal levels of physical activity. Anthropometric measurements and blood sampling were done at the beginning and end of the study period.

**Results:**

CQR-300 as well as CORE exhibited antioxidant properties *in vitro*. They also acted as *in vivo *antioxidants, bringing about significant (p < 0.001) reductions in plasma TBARS and carbonyls. Both CQR-300 and CORE also brought about significant reductions in weight, body fat, total cholesterol, LDL-cholesterol, triglycerides, and fasting blood glucose levels over the respective study periods. These changes were accompanied by a significant increase in HDL-cholesterol levels, plasma 5-HT, and creatinine.

**Conclusion:**

CQR-300 (300 mg daily) and CORE (1028 mg daily) brought about significant reductions in weight and blood glucose levels, while decreasing serum lipids thus improving cardiovascular risk factors. The increase in plasma 5-HT and creatinine for both groups hypothesizes a mechanism of controlling appetite and promoting the increase of lean muscle mass by *Cissus quadrangularis*, thereby supporting the clinical data for weight loss and improving cardiovascular health.

## Background

The incidence of obesity in adults as well as children is on an increase globally. Once considered a problem of developed countries, this global epidemic also affects developing countries. Coupled to this epidemic are obesity-related complications such as cardiovascular disease, stroke, depression, and Type-2 diabetes, which are spreading rapidly across poor and middle-income countries, where infectious diseases and malnutrition have previously overshadowed such illnesses [1]. Obesity is a principal causative factor of metabolic syndrome [2-6]. The coexistence of these metabolic syndromes (hyperglycemia, dyslipidemia, and hypertension in the same individual) is a growing medical problem in industrialized countries [6-9]. It has been reported that obesity may induce systemic oxidative stress and that increased oxidative stress in accumulated fat is associated with dysregulation of adipocytokines and development of metabolic syndrome [6]. Oxidative stress has been shown to be involved in the process of atherogenesis [10], ischemic heart disease [11], obesity [6], metabolic syndrome or syndrome X, diabetes [12,13], as well as in immunodeficiency [14]. The stability of tissues against oxidative stress is however enhanced by antioxidant compounds, which could be present in the diet [15]. Antioxidants have also been shown to be very effective in inhibiting the oxidation of LDL and scavenging of free radicals and reactive oxygen species *in vitro *[16-20]. These compounds are present in fruits and vegetables, which contain natural antioxidants, and are known to delay the onset of atherogenesis.

*Cissus quadrangularis *(Linn) has been used by common folk in India for promoting the fracture healing process. It has been prescribed in Ayurveda as an alterative, anthelmintic, dyspeptic, digestive, tonic, analgesic in eye and ear diseases, and in the treatment of irregular menstruation and asthma. In Cameroon, the whole plant is used in oral re-hydration, while the leaf, stem, and root extracts of this plant are important in the management of various ailments. Earlier works on *Cissus quadrangularis *report its effectiveness on the management of obesity and complications associated with metabolic syndrome [21], as well as its antioxidant and free radical scavenging activity *in vitro *[22,23]. Various formulations now contain extracts of *Cissus quadrangularis *in combination with other compounds, used for the purpose of management of overweight and obesity, as well as complications resulting from these conditions, notably metabolic syndrome (syndrome X). Phytochemical analyses of *Cissus quadrangularis *revealed high contents of ascorbic acid, carotene, anabolic steroidal substances, and calcium. The stem contains two asymmetric tetracyclic triterpenoids, and two steroidal principles. The presence of β-sitosterol, δ-amyrin, δ-amyrone, and flavanoids (quercetin) has also been reported [24,25], all these components having potentially different metabolic and physiologic effects.

Although different uses of *Cissus quadrangularis *have been investigated, [26,27] the antioxidant potential of various *Cissus quadrangularis *formulations have not been evaluated in the modulation of obesity-induced oxidative stress. It was for this reason that the present study was designed, using a *Cissus quadrangularis *formulation (CORE) and CQR-300 (a standardized extract of *Cissus quadrangularis*, 2.5 % keto-steroids, and 15% soluble plant fiber).

## Methods

The study was approved by the Cameroon National Ethics Board. The purpose, nature, and potential risks of the study were explained to all participants, who gave their written informed consent before participation. The study was conducted in accordance with the Helsinki Declaration (1983 version).

### Sample

CORE (Table 1) was obtained from Soy Labs, LLC., Fairfield, California, USA;. CQR-300, a standardized extract of *Cissus quadrangularis *containing 2.5% keto-steroids and 15% soluble plant fiber (Gateway Health Alliances, Inc, Fairfield, California, USA).

**Table 1:** Detailed composition of the cissus formulation (CORE).

Item	Description	Active Amount Per Capsule (mg)	Amount of Active Per Day (2 capsules) (mg)
*Cissus quadrangularis*	5% ketosteroids	7.5	15
ChromeMate*	Niacin-Bound Chromium (10% Cr) – Concentrate	0.15	0.3
Green Tea Extract (High Caffeine)	40% Polyphenols, 22% EGCG, 40% Caffeine	100	200
Selenium	0.5% l-Selenomethionine	0.06	0.12
AlbumaSoy**	Soy Albumin	50	100
Vitamin B6	pyridoxine hydrochloride	50	100
Vitamin B12	cyanocobalamin	0.05	0.1
Folic Acid	Folic acid	0.4	0.8

**Total Per Unit**		**104.08**	**265.645**

*ChromeMate is a trademark of InterHealth N.I.

**AlbumaSoy is trademark of Soy Labs, LLC

### *In vitro *antioxidant potential of the CORE formulation and CQR-300

Both CORE and CQR-300 were dissolved in acidified methanol prepared as previously described by Agbor et al. [19] for the *in vitro *antioxidant study. Three methods were used for the determination of antioxidant potential: Folin (polyphenol content), ferric reducing antioxidant power (FRAP, antioxidant power), 1,1-Diphenyl-2-picrilhydrazyl (DPPH, redical scavenging potential).

#### Polyphenol content

The Folin Ciocalteu reagent (Sigma Chemical Co., St Louis, MO, USA) was used to determine the concentration of polyphenol as a measure of antioxidant potential of CORE and CRQ-300. The reagent was diluted 10 times before used as described by Singleton et al. [28]. The absorbance was measured at 750 nm (Genesys Spectronic 20).

Ferric Reducing Antioxidant Power (**FRAP**) was measured as earlier described by Benzie and Strain. [29]. In brief, 2000 μl of freshly prepared FRAP reagent (10 parts of 300 mM acetate buffer (pH 3.6), 1 part of 10 mM TPTZ (Sigma, in 400 mM HCL), and 1 part of 20 mM ferric chloride). After an initial incubation for 15 minutes at 37°C, the absorbance was read at 593 nm.

Scavenging potential against 1,1-Diphenyl-2-picrilhydrazyl (**DPPH**) measured the ability of the extracts to scavenge free radicals. 20 μl of extract was introduced into 2 ml methanolic solution of DPPH (0.3 mM) and kept in the dark for 30 minutes. The extract was replaced by methanol for the control, and catechin was used as the standard. The absorbance was read at 517 nm, and the antioxidant content and percentage inhibition of the extract calculated as earlier described by Yen and Duh, [30].

In all three methods mentioned above, measurements were done in triplicates.

### *In vivo *study

#### Participants

A total of 168 overweight, obese, and normal weight participants aged between 19 and 50 years were selected from a group responding to a radio and poster advert. The BMIs of participants ranged from 25.0 to 48.7, and their weights ranged from 70.6 to 142 kg. After physical examination, which included measurement of blood pressure, participants with unusually elevated fasting blood glucose levels, those who were pregnant or lactating, as well as those on any form of weight-reducing medication were excluded from the study. Also excluded were participants who were involved in intense exercise programs, had medical conditions known to affect serum lipids, or had a history of drug or alcohol abuse. A total of 153 participants completed the study, while 15 participants dropped out of the study for personal reasons (7 moved out of town or had to travel during study period; 4 participants thought they had lost enough weight; while 4 participants were on malaria treatment and were excluded before the end of the study period).

### Trial protocol

The study was double-blind placebo controlled, with the 168 overweight, obese, or normal weight participants of both sexes (between 19 and 50 years) distributed as outlined in Table 2. The participants who were either on their normal diet or on an energy restricted (2100 Kcal/day) diet, received two daily doses in the form of capsules of CORE for 8 weeks, and CQR-300 or placebo for 6 weeks. The capsules were identical in shape, color, and appearance, with neither the participants nor researchers knowing what capsule they received. Side effects were solicited on each weekly visit. Body weight and percentage body fat were determined in 12-hour fasted participants using a Tanita™ scale. Height was measured with a stadiometer to the nearest 0.5 cm. Blood samples were obtained at the start and end of the trial period after a 12-hour overnight fast, into heparinized tubes, for the measurement of LDL oxidation (TBARS) [31] and protein carbonyl [32] content, total cholesterol, triacylglycerol, HDL-cholesterol, LDL-cholesterol, and glucose (cholesterol Infinity, triglyceride Infinity, EZ HDL™ cholesterol, EZ LDL™ cholesterol, Glucose Trinder) from SIGMA Diagnostics. Serotonin was measured using an enzyme immunoassay method (Serotonin EIA kit, BioSource Europe S.A, Belgium), creatinine by a modification of the method of Bartels et al. [33], while MDA was measured by a standard established method [34].

**Table 2:** Distribution of participants into treatment groups. The number given in parentheses represents the participants whose complete measurements were done over the study period.

Group No.	Participants	Treatment	No. of Participants
1	Males/Females BMI>30	Placebo 6 weeks (2100 Kcal/day diet)	33 (32)
2	Males/Females BMI>30	CORE 8 weeks (no dietary restriction)	33 (30)
3	Males/Females BMI>30	CORE 8 weeks (2100 Kcal/day diet)	35 (31)
4	Males/Females BMI>30	CQR-300 6 weeks (2100 Kcal/day diet)	35 (32)
5	Males/Females BMI 25–29.9	CORE 8 weeks (no dietary restriction)	32 (28)

### Statistical analyses

Statistical Package for the Social Sciences (SPSS) [35] software was used for all statistical analysis. The data were presented as means ± SD. The statistical difference between samples was assessed by a Student's t-test for normal distribution or the Mann-Whitney test for non-normal distribution, after ANOVA testing of all the groups showed that significant differences existed. Paired Student's t-test was carried out on the start and end values of all the groups.

## Results

### *In vitro *fantioxidant potential of CORE and CQR-300

The *in vitro *antioxidant capacity of CORE was significantly (p < 0.01) higher than that of CQR-300 irrespective of the method of analysis used. Considering the composition of CORE (Table 1), it is likely that the other components present act synergistically with the *Cissus quadrangularis *extract present (Table 3).

**Table 3:** *In vitro *antioxidant capacity of the CORE and CQR-300 (mg of catechin equivalent/gram dry weight) by FRAP, Folin, and DPPH methods.

	FRAP Antioxidant (mg/g)	Folin Total Polyphenol (mg/g)	DPPH Antioxidant (mg/g)
CORE	22.67 ± 4.58	56.70 ± 6.12	8.46 ± 1.30
CQR-300	15.85 ± 3.08*	42.33 ± 3.21*	5.97 ± 0.66*

*p < 0.01 compared to the cissus formulation for each method.

Table 4 presents the effect of CORE and CQR-300 on the oxidative stress parameter (TBARS and carbonyls). CORE was more effective in reducing oxidative stress than CQR-300, the reduction being more obvious in the diet-restricted group. It significantly (p < 0.01) reduced the formation of TBARS and carbonyls compared to CQR-300.

**Table 4:** The effect of the CORE and CQR-300 extracts on the concentrations of TBARS and carbonyls.

	Group 1 BMI>30 Placebo (2100 Kcal/day diet)		Group 2 BMI>30 CORE (no dietary restriction)		Group 3 BMI>30 CORE (2100 Kcal/day diet)		Group 4 BMI>30 CQR-300 (2100 Kcal/day diet)		Group 5 BMI 25–29.9 CORE (no dietary restriction)	
Time (weeks)	T = 0	T = 6	T = 0	T = 8	T = 0	T = 8	T = 0	T = 6	T = 0	T = 8
TBARS (umol/L)	1.42 ± 0.51	1.60 ± 0.38	1.66 ± 0.56	0.87 ± 0.53**	1.78 ± 0.38	0.72 ± 0.35**^†^	1.84 ± 0.51	0.98 ± 0.18**	1.06 ± 0.24	0.66 ± 0.13**^†^
Carbonyls (nmol/mg protein)	1.95 ± 0.73	2.09 ± 0.68	2.03 ± 0.39	0.98 ± 0.39**	1.96 ± 0.40	0.84 ± 0.27**^†^	2.06 ± 0.28	1.10 ± 0.34**	0.94 ± 0.20	0.77 ± 0.36**^†^

**p < 0.001 compared to T = 0; ^†^p < 0.01 compared to CQR-300 (Group 4).

### Effect of CORE and CQR-300 on body weight

Obese participants, who received CQR-300 (300 mg daily), had significantly (p < 0.05) greater reduction in body weight compared to those on placebo (Table 5). This reduction in body weight corresponded to a 5.4 % reduction in BMI. CORE had a more significant (p < 0.01) effect (8.5% reduction for participants on an energy restricted diet) on the weight of participants compared to CQR-300. There was no significant net change in weight in participants on placebo during the study period.

**Table 5:** Effect oftheCORE and CQR-300 on body weight, BMI, and Body fat

	Group 1 BMI>30 Placebo (2100 Kcal/day diet)		Group 2 BMI>30 CORE (no dietary restriction)		Group 3 BMI>30 CORE (2100 Kcal/day diet)		Group 4 BMI>30 CQR-300 (2100 Kcal/day diet)		Group 5 BMI 24–29.9 CORE (no dietary restriction)	
Time (weeks)	T = 0	T = 6	T = 0	T = 8	T = 0	T = 8	T = 0	T = 6	T = 0	T = 8
Weight (kg)	112.4 ± 2.6	113.6 ± 2.0 (1.1)	95.8 ± 11.8	89.2 ± 9.2 (6.9)*	95.3 ± 14.6	87.2 ± 8.9 (8.5)**^†^	118.6 ± 3.8	113.8 ± 2.5 (4.0)*	76.3 ± 6.8	72.5 ± 4.7 (5.0)*
BMI (kg/m^2^)	38.1 ± 1.1	38.0 ± 0.9	37.7 ± 6.3	34.6 ± 8.6	37.5 ± 4.7	33.8 ± 6.9	38.8 ± 1.0	36.7 ± 3.4	27.3 ± 2.5	26.3 ± 3.0
Body fat (%)	43.6 ± 1.6	42.8 ± 2.1	46.5 ± 3.1	43.7 ± 2.4	48.1 ± 4.4	44.3 ± 2.1	44.3 ± 5.4	42.1 ± 3.6	35.9 ± 2.2	34.2 ± 3.0

Values are means ± SE.

*p < 0.05, **p < 0.01, compared to placebo,^†^p < 0.01 compared to CQR-300 (Group 4), () = percentage change in weight.

Table 6 presents the effect of CORE and CQR-300 on blood lipids and fasting blood glucose levels. For participants on a restricted diet, six weeks use of CQR-300 reduced plasma total cholesterol by 18.0%, LDL-cholesterol by 29.0%, triacylglycerol by 21.7%, and fasting blood glucose by 14.6%. This treatment also increased the concentration of HDL-cholesterol by 21.1%. On the other hand, CORE (group 3) reduced the concentration of plasma total cholesterol by 26.0%, LDL-cholesterol by 32.4%, triacylglycerol 28.0%, and fasting blood sugar 16.1%. The CORE formulation also increased HDL cholesterol by 43.0%. The above mentioned changes were less obvious in participants whose diets were not restricted.

**Table 6:** Effect of the CORE and CQR-300 on blood lipids and fasting blood glucose

	Group 1 BMI>30 Placebo (2100 Kcal/day diet)		Group 2 BMI>30 CORE (no dietary restriction)		Group 3 BMI>30 CORE (2100 Kcal/day diet)		Group 4 BMI>30 CQR-300 (2100 Kcal/day diet)		Group 5 BMI 24–29.9 CORE (no dietary restriction)	
Time (weeks)	T = 0	T = 6	T = 0	T = 8	T = 0	T = 8	T = 0	T = 6	T = 0	T = 8
Total Cholesterol (mg/dL)	136.3 ± 9.0	138.3 ± 11.1	159.1 ± 14.6	116.2 ± 9.7*	171.0 ± 15.9	126.5 ± 7.9 *^†^	138.3 ± 13.8	113.4 ± 4.5*	152.6 ± 8.8	123.0 ± 4.7*
LDL-cholesterol (mg/dL)	88.4 ± 5.9	88.2 ± 5.9	99.8 ± 6.5	81.4 ± 1.7	116.6 ± 5.4	78.8 ± 3.3	92.7 ± 7.0	65.8 ± 3.4	101.6 ± 1.9	74.8 ± 0.9
HDL-cholesterol (mg/dL)	27.3 ± 2.2	25.8 ± 3.1	36.6 ± 4.7	55.1 ± 6.4*	38.6 ± 10.5	55.2 ± 8.6*^†^	25.6 ± 4.8	31.0 ± 3.9	44.4 ± 7.7	52.0 ± 8.1
Triacylglycerol (mg/dL)	93.6 ± 4.8	90.5 ± 10.4	156.0 ± 16.8	95.6 ± 8.1	144.9 ± 43.3	104.3 ± 26.2*^†^	93.8 ± 9.7	73.4 ± 6.0***	117.4 ± 9.8	99.8 ± 11.2
Fasting Blood glucose (mg/dL)	93.6 ± 7.2	89.4 ± 10.1	101.3 ± 8.6	87.7 ± 10.0	102.4 ± 1.8	85.9 ± 5.2	91.8 ± 6.9	78.4 ± 11.2	93.3 ± 10.2	82.7 ± 7.2

Values are means ± SE, comparing starting point to end point, significant differences were at *p < 0.05, **p < 0.01 and ***p < 0.001 for the same treatment.

The effect of CORE and CQR-300 on malondialdehyde (MDA), serum serotonin, and creatinine levels are presented in Table 7. CQR-300 significantly (p < 0.05) reduced the concentration of plasma MDA. This effect was accompanied by a slight increase in the urinary concentration of MDA though not significant. CQR-300 also significantly (p < 0.05) increased the concentration of plasma 5-HT by 53.3% and plasma creatinine levels by 23.5%. An increase in 5-HT of 17.0% was also observed in the placebo group, while CORE (group 3) showed a significant increase of 39.1%. As such results for 5-HT and creatinine were significantly (p < 0.05) lower for CORE than CQR-300.

**Table 7:** Effect of the CORE andCQR-300 on plasma and urinary malondialdehyde, 5-HT, and plasma creatinine levels

	Group 1 BMI>30 Placebo (2100 Kcal/day diet)		Group 2 BMI>30 CORE (no dietary restriction)		Group 3 BMI>30 CORE (2100 Kcal/day diet)		Group 4 BMI>30 CQR-300 (2100 Kcal/day diet)		Group 5 BMI 24–29.9 CORE (no dietary restriction)	
Time (weeks)	T = 0	T = 6	T = 0	T = 8	T = 0	T = 8	T = 0	T = 6	T = 0	T = 8
Plasma MDA (μmol/L)	1.8 ± 0.1	1.7 ± 0.1	1.8 ± 0.6	1.0 ± 0.5*	1.9 ± 0.9	1.1 ± 0.6 *^†^	1.6 ± 0.2	1.0 ± 0.4*	1.8 ± 0.3	1.2 ± 0.5*
Urinary MDA (μmol/day)	3476.2 ± 211.7	3492.2 ± 188.4	3864.3 ± 238.4	4325.1 ± 167.5*	4022.1 ± 148.1	4485.8 ± 203.3	3416.2 ± 110.9	3581.5 ± 200.2	3593.8 ± 116.5	3971.0 ± 104.5
Plasma 5-HT (mgl/dl)	30.6 ± 1.9	35.8 ± 2.1	32.5 ± 1.9	42.4 ± 3.4*	38.4 ± 1.5	53.4 ± 3.6^†^	35.4 ± 1.7	54.3 ± 2.9*	33.1 ± 3.7	43.2 ± 2.1*
Plasma creatinine (mg/dl)	25.7 ± 3.8	27.3 ± 1.4	25.9 ± 1.8	31.8 ± 2.1	24.4 ± 3.2	28.9 ± 1.2	27.2 ± 2.2	33.6 ± 1.4*	29.0 ± 0.8	33.8 ± 1.2

Values are means ± SD, comparing starting point to end point, significant differences were at *p < 0.05 for the same treatment.

## Discussion

The role of antioxidants from natural products in degenerative disease has attracted more interest on natural products research. In this study, we evaluated the antioxidant potential of CORE and CQR-300 on obesity-induced oxidative stress. The parent plant in these two formulations is *Cissus quandrangularis*. The formulation had a higher *in vitro *antioxidant potential than CQR-300 irrespective of the method used for the assay. The high antioxidant potential of CORE may be due to its composition. It contains some tea polyphenols and selenium that are potential antioxidants and thus complement the antioxidant potential of the parent plant in this formulation (*Cissus quandrangularis*). On the other hand, CQR-300 is a standardized extract of *Cissus quandrangularis *and no antioxidant was added to it.

Obesity may induce systemic oxidative stress, and increased oxidative stress in accumulated fat is one of the underlying causes of dysregulation of adipocytokines and development of metabolic syndrome [6]. Oxidative stress plays critical roles in the pathogenesis of various diseases [36]. In order to investigate if oxidative stress was increased in the obese participants, we measured lipid peroxidation (which represent the plasma TBARS) and the carbonyl compounds as markers of oxidative injury, which correlates with the BMI. The high plasma concentration of TBARS and carbonyl compounds was an indication of oxidative stress in the obese and overweight participants. These concentrations were significantly (p < 0.01) reduced after treatment, with CORE being more effective than CQR-300. These samples may function through two mechanisms: either by scavenging free radicals to reduce oxidative stress, or by clearing the plasma of the products that are themselves potential oxidants. These activities may be attributed to the polyphenols present in the different formulations.

The use of CORE and CQR-300 during the study period brought about a significant reduction in the weight and BMI of obese patients. This loss in weight was comparable to that observed with cissus studies [21], sibutramine for one year [37], and orlistat for 6 months or 1 year [38,39]. This reduction in BMI was accompanied by an increase in HDL-cholesterol, and corroborates earlier work that showed an inverse relation between BMI and HDL-cholesterol, the latter imparting possible health benefits in overweight and obese people [40,41]. The increase in the concentration of HDL-cholesterol and a decrease in the concentration of LDL-cholesterol could lead to a lowering of the atherogenicity and therefore a significant reduction in the potential incidence of coronary heart disease [42] (54% reduction of risk for a 0.6 mmol/L reduction of serum cholesterol) [43]. A reduction of fasting blood glucose levels as well as MDA levels have been previously reported to accompany weight loss in obese subjects [39]. The above observation could be linked to an increase in circulating creatinine and serotonin over the eight-week trial period. Serotonin is known to have a positive effect on mood and to reduce binge eating, which is common in obese people. Several previous studies [44,45] have shown a direct link between serotonin levels and weight loss. On the other hand, an increase in creatinine concentrations parallels an increase in lean muscle mass and a probable reduction in body fat.

Furthermore, *in vitro *studies (submitted in a different publication) show the ability of *Cissus quadrangularis *extracts to inhibit pancreatic lipase by approximately 60%, alpha-amylase by approximately 90%, as well as alpha-glucosidase by approximately 39%, all of which could contribute to weight reduction in obesity.

## Conclusion

The CORE and CQR-300 (300 mg daily) brought about a significantly greater weight loss than placebo during the study period in obese individuals. This was accompanied by a significant improvement in the lipid profiles, blood sugar profiles, and serotonin profiles of study participants. They could have additional properties as antioxidants against oxidative stress in obese individuals. Thus, CQR-300 as well as CORE possesses antioxidant and free radical scavenging properties that could have applications in metabolic as well as other physiological complications in which there is an increase in oxidative stress.

These new findings warrant further exploration into the active phytonutrients of *Cissus quadrangularis *and the potential of its newly discovered weight loss and cardiovascular health benefits.

## Authors' contributions

JO conceived, designed, and coordinated the work, as well as drafted the manuscript; DM and GF carried out analytical work; YS carried out analytical and statistical analyses of data; GA participated in the design and editing of the manuscript.

All authors have read and approved the manuscript.
